# Causal effects of opioids on postpartum depression: a bidirectional, two-sample Mendelian randomization study

**DOI:** 10.3389/fpsyt.2023.1043854

**Published:** 2023-04-20

**Authors:** Yage Jiang, Donglei Wei, Yubo Xie

**Affiliations:** ^1^Department of Anesthesiology, The First Affiliated Hospital of Guangxi Medical University, Nanning, China; ^2^Department of Orthopedic Trauma and Hand Surgery, The First Affiliated Hospital of Guangxi Medical University, Nanning, China; ^3^Guangxi Key Laboratory of Enhanced Recovery after Surgery for Gastrointestinal Cancer, The First Affiliated Hospital of Guangxi Medical University, Nanning, China

**Keywords:** postpartum depression, opioids, analgesics, Mendelian randomization, single nucleotide polymorphisms

## Abstract

**Background:**

Postpartum depression is the most common psychiatric disorder in pregnant women during the postpartum period and requires early detection and treatment. Previous studies have found that opioids use affects depression and anxiety disorders. Although it has long been suspected that opioids may contribute to the development of postpartum depression, observational studies are susceptible to confounding factors and reverse causality, making it difficult to determine the direction of these associations.

**Methods:**

To examine the causal associations between opioids and non-opioid analgesics with postpartum depression, we utilized large-scale genome-wide association study (GWAS) genetic pooled data from two major databases: opioids, salicylate analgesic, non-steroidal anti-inflammatory drugs (NSAIDs), and aniline analgesics GWAS data from the United Kingdom Biobank database. GWAS data for postpartum depression were obtained from the FinnGen database. The causal analysis methods used random-effects inverse variance weighting (IVW), and complementary sensitivity analyses using weighted median, MR-Egger method, and MR-PRESSO test.

**Results:**

In the IVW analysis, Mendelian randomization (MR) analysis showed that opioids increased the risk of postpartum depression (OR, 1.169; 95% CI, 1.050–1.303; *p* = 0.005). Bidirectional analysis showed a significant causal relationship between genetically predicted postpartum depression and increased risk of opioids and non-opioid analgesics use (opioids OR, 1.118; 95% CI, 1.039–1.203; *p* = 0.002; NSAIDs OR, 1.071; 95% CI, 1.022–1.121; *p* = 0.004; salicylates OR, 1.085; 95% CI, 1.026–1.146; *p* = 0.004; and anilides OR, 1.064; 95% CI, 1.018–1.112; *p* = 0.006). There was no significant heterogeneity or any significant horizontal pleiotropy bias in the sensitivity analysis.

**Conclusion:**

Our study suggests a potential causal relationship between opioids use and the risk of postpartum depression. Additionally, postpartum depression is associated with an increased risk of opioids and non-opioid analgesics use. These findings may provide new insights into prevention and intervention strategies for opioids abuse and postpartum depression.

## 1. Introduction

Postpartum depression (PPD) is a common psychiatric disorder of the puerperium with clinical manifestations of mood changes, depressed attitudes to life, and mental and psychiatric changes, with suicidal tendencies in severe cases (1, 2). The symptoms of PPD begin 4–6 weeks after delivery and peak 2–3 months after birth, with onset ranging from a few weeks to as long as 12 months after delivery (3, 4). Most patients recover from PPD within a few months, but approximately 25–30% continue to experience PPD 1 year after delivery (5). Lindahl et al. estimated pregnant and postpartum suicide rates from published and unpublished reports globally. Suicide accounts for up to 20% of postpartum deaths, while 5–14% of pregnant and postpartum women have suicidal thoughts (6). Depression, intimate partner violence, and substance use disorders are the three most common risk factors for pregnancy-related suicide (7). All of these have negative effects on the mother, the child, and the entire family and impose considerable social costs.

The opioid epidemic has received significant attention in recent years, especially in the perinatal period due to the use of opioids. Inadequate treatment of postpartum pain has a significant impact on both women and their families. Unlike patients who return to activities independently for other procedures, most women who undergo cesarean delivery must immediately care for their newborn and other family members. Therefore, the potential benefits and harms associated with opioid exposure need to be carefully evaluated prior to using opioids for such pain management. Although these figures vary by geographic location and setting, it is believed that more than 80% of women utilize opioid prescription drugs following cesarean delivery and around 54% after vaginal delivery (8). During their reproductive years, however, all women, especially those aged 18–29, are at risk for substance use disorders, which may be associated with hormonal changes during the menstrual cycle, delivery, pregnancy, lactation, and menopause (9, 10). On the other hand, Benningfield et al. discovered that 64.6% of 174 opioid-dependent pregnant women were diagnosed with at least one mental health condition, including 40% for anxiety, 33% for severe depression, and 48.8% for mood disorders (11). 77% of pregnant women who died of a drug overdose had a mental health condition, according to another study (12).

However, determining the causal effect of opioids on postpartum depression is challenging, mainly due to the inability to avoid confounding factors and reverse causality. Mendelian randomization (MR) studies are a new way to look for possible causes, and using genetic variation as an instrumental variable (IV) can reduce bias caused by confounders and reverse causality (13). Therefore, this study investigated potential causal associations of opioids and three non-opioid analgesics with postpartum depression by a bidirectional MR analysis using pooled data from two GWAS studies (United Kingdom Biobank and FinnGen database).

## 2. Materials and methods

### 2.1. Opioids and non-opioid analgesics

Pooled GWAS data for opioids and three non-opioid analgesics were obtained from a large meta-analysis by the United Kingdom Biobank (UKB) (14). The study included 502,616 participants (approximately 54% female) with a mean age of 56.53 (standard deviation of 8.09) years and a mean body mass index (BMI) of 27.43 (standard deviation of 4.80). The proportion of subjects taking medications increased with age. The Anatomical Therapeutic Chemical Classification System was used to divide analgesic drugs into 23 groups based on their active ingredients. Some of these groups contain opioids (like morphine, oxycodone, codeine, fentanyl, pethidine, and tramadol), nonsteroidal anti-inflammatory drugs (NSAIDs), anilides, and salicylic acid derivatives (14). As the number of SNPs that meet 5 × 10–8 is small, it is difficult to identify enough SNPs to use in an instrumental variable analysis. Therefore, we screened all relevant single nucleotide polymorphisms (SNPs) in the GWAS data based on the selection threshold *p* value of less than 5 × 10–6 and unrelated condition (10,000 kbp apart and *R*^2^ ≤ 0.001). These SNPs’ associations with exposure factors were evaluated using the F statistic (F = beta^2^/se^2^), and low-statistical-power SNPs will be excluded (F statistic <10).

### 2.2. Postpartum depression

To reduce sample overlap, we extracted summary statistics for postpartum depression from the FinnGen database (https://r7.finngen.fi/, Public release: June 1, 2022). A dataset that followed women who delivered between 1998 and 2019, and contained 226,707 (11,711 cases and 214,996 controls) participants with a mean age at the first event of 40.89 years, with screening conditions including mild mental and behavioral disorders associated with the puerperium, recurrent depressive disorders, and depressive episodes. As previously described, we used independently aggregated SNPs that met thresholds (*p* < 5 × 10–6, *F* > 10, *R* ^2^ < 0.001 and 10,000 kbp apart) as instrumental variables.

### 2.3. MR analysis

**Figure 1:**
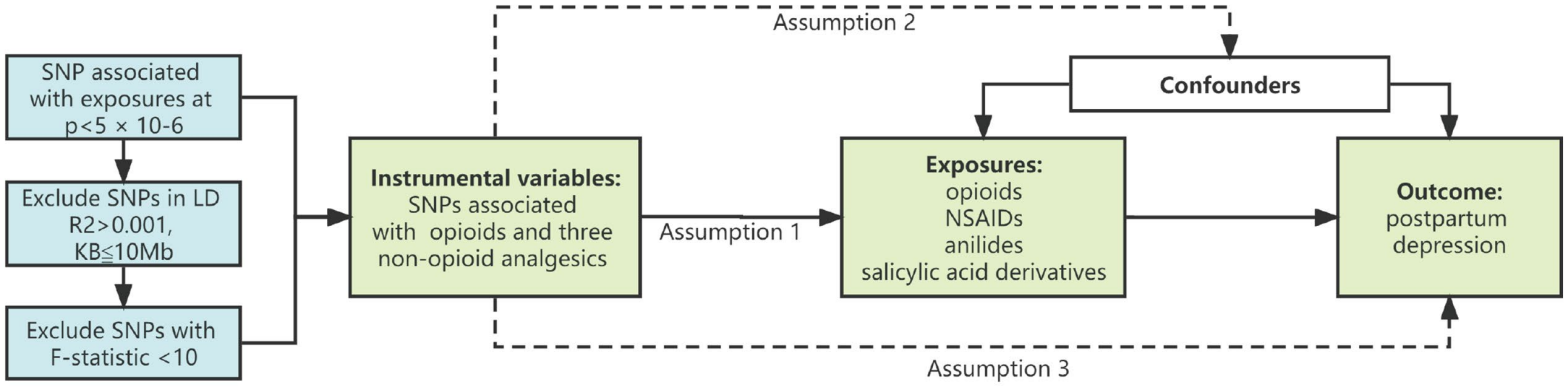
Study design diagram and three assumptions of Mendelian randomization. (1) Genetic variation is closely related to exposure; (2) Genetic variation is not related to any potential confounding factors; (3) Genetic variation is not related to the outcome unless through the mode of exposure. SNP, single nucleotide polymorphism; NSAIDs, nonsteroidal anti-inflammatory drugs.

The “TwoSampleMR” package was utilized in R (version 4.1.2) for the purpose of conducting Mendelian randomization analysis. A summary of the study design is shown in Figure 1, showing the three main assumptions of the MR study. In this investigation, three different MR approaches were carried out for each of the possible directions of the impact between postpartum depression and the risk of opioids and non-opioid analgesics. The traditional inverse variance weighting (IVW) is the most effective method for genetically predicting the association (15). To determine reverse causation, the study was bidirectional. In addition, weighted median and MR-Egger regression methods were employed to eliminate bias induced by horizontal pleiotropy and to assess the robustness of the IVW method (16). When no genetic variant was found to be a valid instrumental variable, the MR Egger regression intercept and 95% confidence interval (CI) were used to assess the degree of bias in the casual estimates due to directional pleiotropy (16, 17). When 50% of genetic variants are valid IVs, the weighted median gives a consistent evaluation of causative effects (18). Sensitivity analyses used “leave one out analysis” to explore the possibility of a single SNP driving this causal association. The MR-egger intercept and MR PRESSO methods were used to detect the presence of horizontal pleiotropy (19). If outliers are detected by the MR-PRESSO method, they will be removed, and re-evaluate the MR causality.

For binary outcomes, the odds ratio (OR) and 95%CI was applied to estimate the degree of causality. In addition, in the MR analysis, we tested the effect of four exposures on the outcome. Therefore, we used a Bonferroni-corrected *p* value (*p* < 0.05/4 = 0.0125) as the threshold for statistical significance.

### 2.4. Ethical approval and data availability

This study did not require ethical approval from an institutional review board because the pooled data were gathered from published studies whose respective institutional review boards had granted approval.

## 3. Results

### 3.1. Genetic instrumental variables for postpartum depression, opioids, and non-opioid analgesics

There were 25, 31, 37, 32, and 37 independent variants for postpartum depression, opioids, NSAIDs, salicylates, and anilides, respectively. Genetic tools for each SNP used for postpartum depression, opioids, NSAIDs, salicylates, and anilides are listed in Supplementary Tables 1–5 (GWAS significance, *p* < 5 × 10–6, F-statistic >10, *R*^2^ < 0.001, and greater than 10 Mb; Table 1).

**Table 1:** MR results of opioids and non-opioid analgesics on risk of postpartum depression.

Exposures	OR (95%CI)	Pval	Used of SNPs	Cochrane’s Q	Heterogeneity pval	Pleiotropy pval
Opioids						
MR Egger	1.059 [0.766, 1.465]	0.729	31	38.35	0.115	0.531
Weighted median	1.192 [1.041, 1.364]	0.011^*^				
Inverse variance weighted	1.169 [1.050, 1.303]	0.005^*^				
MR-PRESSO	1.169 [1.050, 1.303]	0.008^*^				
NSAIDs						
MR Egger	1.080 [0.648, 1.802]	0.768	37	42.11	0.19	0.853
Weighted median	1.034 [0.850,1.256]	0.740				
Inverse variance weighted	1.031 [0.895, 1.189]	0.671				
MR-PRESSO	1.031 [0.895, 1.189]	0.673				
Salicylic acid and derivatives						
MR Egger	1.353 [1.005, 1.823]	0.056	32	28.35	0.552	0.161
Weighted median	1.174 [0.993, 1.387]	0.060				
Inverse variance weighted	1.106 [0.987, 1.239]	0.083				
MR-PRESSO	1.106 [0.988, 1.238]	0.090				
Anilides						
MR Egger	1.077 [0.615, 1.885]	0.797	37	40.63	0.236	0.727
Weighted median	1.272 [1.042, 1.553]	0.018				
Inverse variance weighted	1.187 [1.026, 1.373]	0.022				
MR-PRESSO	1.187 [1.026, 1.373]	0.025				

MR, mendelian randomization; OR, odds ratio; SNP, single nucleotide polymorphism; and NSAIDs, nonsteroidal anti-inflammatory drugs. * Indicates significant difference, less than Bonferroni-corrected *p* value (*p*<0.0125).

### 3.2. Effects of opioids and non-opioid analgesics on postpartum depression

**Figure 2:**
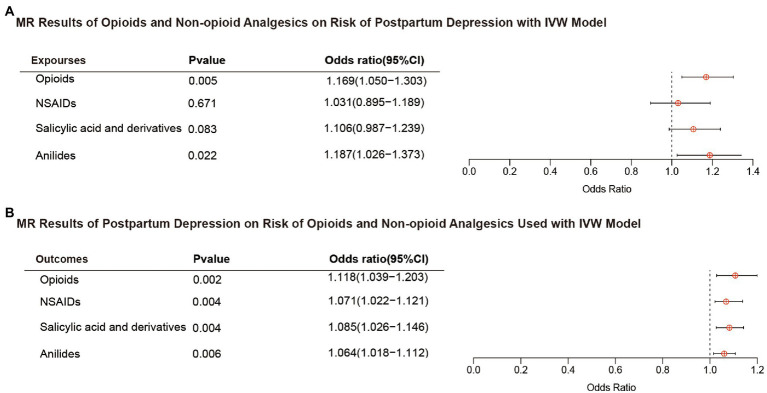
Results of bidirectional MR analysis with IVW model. **(A)** Opioids and non-opioid analgesics on risk of postpartum depression. **(B)** Postpartum depression on risk of opioids and non-opioid analgesics used. MR, mendelian randomization; IVW, inverse variance weighted; OR, odds ratio; and NSAIDs, nonsteroidal anti-inflammatory drugs.

**Figure 3:**
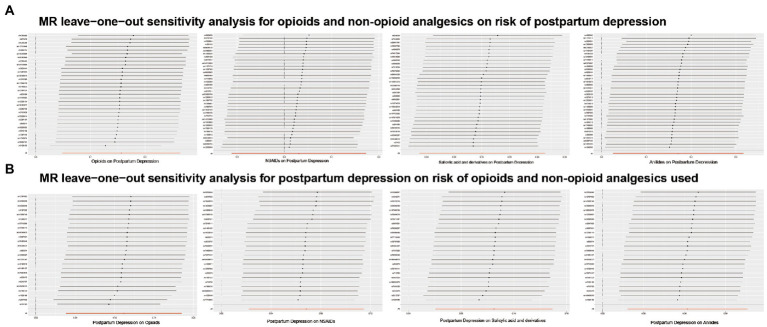
Results of MR leave-one-out sensitivity analyses. **(A)** Opioids and non-opioid analgesics on risk of postpartum depression. **(B)** Postpartum depression on risk of opioids and non-opioid analgesics used.

Figure 2A shows the association between opioids and non-opioid analgesics with postpartum depression. We found a significant causal relationship between the use of opioids and the increased risk of postpartum depression, and the IVW OR was 1.169 [95% CI, 1.050–1.303; *p* = 0.005]. No significant causal relationship found between non-opioid analgesics and postpartum depression. Cochran Q statistics showed no significant heterogeneity in SNP effects, and the results of MR-Egger intercept tests did not show any significant horizontal pleiotropy bias. In addition, leave-one-out analyses revealed that instrumental variables did not influence causal inference, and their MR results were robust (Figure 3A).

### 3.3. Effect of postpartum depression on opioids and non-opioid analgesics use

Figure 2B shows the results of the reverse MR analysis, where we found a significant causal relationship between genetically predicted postpartum depression with the risk of opioids and non-opioid analgesics use (*p* < 0.0125). Postpartum depression was likely to increase the risk of opioids and non-opioid analgesics use (opioids OR, 1.118; 95% CI, 1.039–1.203; *p* = 0.002; NSAIDs OR, 1.071; 95% CI, 1.022–1.121; *p* = 0.004; salicylates OR, 1.085; 95% CI,1.026–1.146; p = 0.004; and anilides OR, 1.064; 95% CI,1.018–1.112; *p* = 0.006). Cochran Q statistics for the four analgesics showed no significant heterogeneity in SNP effects (Table 2). The results of the MR-Egger intercept test did not show any significant bias in horizontal pleiotropy (Table 2). In addition, the leave-one-out analysis revealed no instrumental variables affecting causal inference, and their MR results were robust (Figure 3B).

**Table 2:** MR results of postpartum depression on risk of opioids and non-opioid analgesics used.

Outcome	OR (95%CI)	Pval	Used of SNPs	Cochrane’s Q	Heterogeneity pval	Pleiotropy pval
Opioids						
MR Egger	0.957 [0.786, 1.166]	0.666	25	28.80	0.187	0.111
Weighted median	1.042 [0.953, 1.139]	0.368				
Inverse variance weighted	1.118 [1.039, 1.203]	0.002^*^				
MR-PRESSO	1.118 [1.039, 1.203]	0.002^*^				
NSAIDs						
MR Egger	1.054 [0.925, 1.202]	0.438	25	30.76	0.129	0.806
Weighted median	1.087 [1.024, 1.155]	0.006				
Inverse variance weighted	1.071 [1.022, 1.121]	0.004^*^				
MR-PRESSO	1.071 [1.022, 1.121]	0.004^*^				
Salicylic acid and derivatives						
MR Egger	1.058 [0.904, 1.239]	0.488	25	33.80	0.068	0.748
Weighted median	1.077 [1.000, 1.159]	0.049				
Inverse variance weighted	1.085 [1.026, 1.146]	0.004^*^				
MR-PRESSO	1.085 [1.026, 1.146]	0.004^*^				
Anilides						
MR Egger	0.989 [0.876, 1.116]	0.859	25	29.11	0.216	0.177
Weighted median	1.057 [0.998, 1.119]	0.058				
Inverse variance weighted	1.064 [1.018, 1.112]	0.006^*^				
MR-PRESSO	1.064 [1.018, 1.112]	0.006^*^				

MR, mendelian randomization; OR, odds ratio; SNP, single nucleotide polymorphism; and NSAIDs, nonsteroidal anti-inflammatory drugs. * Indicates significant difference, less than Bonferroni-corrected *p* value (*p*<0.0125).

## 4. Discussion

This study systematically assessed the potential bidirectional association between postpartum depression and the risk of opioids or non-opioid analgesics use by the publicly available large-scale GWAS dataset. We found that genetically predicted opioids use was associated with an increased risk of postpartum depression. In addition, reverse MR analysis revealed the potential for postpartum depression to increase the risk of opioids and three non-opioid analgesics use.

In a study of more than 15,000 mother-infant dyads, Faherty et al. found that women with long-term prenatal opioid use had a 1.8-fold higher risk of depression compared to women with no history of opioid use (19). Similarly, prior research has revealed that the use of opioid prescription drugs is connected with an increased risk of serious depression (20). Stigma, social isolation, deteriorating physical health, and a general decline in quality of life may play a crucial moderating role in the association between opiate use and depression (21, 22). These processes are common in people suffering from chronic pain and those who are addicted to opioids. Because the opioid system may be directly engaged in mood regulation, opioid abuse-related dysregulation of the endogenous opioid system may contribute to the development, exacerbation, or maintenance of depression, anxiety, or other mood disorders (23–25). Also, studies in animals and humans have shown that opioids suppress the gonadal axis and that long-term use may lead to hypogonadism (26). Patients with hypogonadism may experience sexual dysfunction and diminished desire, which can have a substantial impact on the development or progression of mood and other mental health problems. The potential mechanisms of opioids in the pathophysiology of depression remain to be elucidated. The κ opioid receptor and its endogenous ligand, prednisolone, have been reported to be strongly associated with stress-induced behavioral deficits (including aversion and irritability) and the development of depression (27). Furthermore, ERK activation was found to play a crucial role in regulating depression and as an essential mediator of depression-like behaviors (28–30). ERK regulates morphine withdrawal and κ opioid receptor activation-induced aversive mood (31, 32). In contrast, systemic κ opioid receptor antagonism can inhibit ERK activity, hence preventing the development of persistent social defeat stress-induced depression (including social avoidance, behavioral despair, and anxiety-related behaviors) (33, 34). Thus, κ opioid receptor-induced ERK activation may be a potential mechanism for opioid-induced depression-like behaviors.

In addition, through reverse MR analysis, the present study identified postpartum depression as a possible causal risk factor for opioids and three pain medication use, similar to previous study’s results (20). Anxious and depressed patients tend to have negative processing biases, especially negative attention and interpretation biases, which may affect pain perception (35). Maternal perinatal anxiety states, as well as depression, have similarly been shown to be associated with higher postpartum pain perception and increased risk of opioids abuse for non-pain symptoms (36, 37). Overprescribing medications is common in women undergoing cesarean delivery and in women who deliver vaginally (8). The appropriate balance for postpartum pain management lies between concerns about under-treatment pain and over-prescribing opioids. Finding this balance requires a comprehensive and objective reassessment of evidence, practice, and preferences (8, 38). Although there are no clear evidence-based recommendations for postpartum pain management, it is strongly advised to weigh the potential benefits against the well-known hazards prior to prudent opioid use. The effectiveness of new approaches to postpartum pain control, such as meditation and breathing exercises, should also be explored for women known to suffer from depression (36).

Furthermore, the present study found that postpartum depression increased the risk of taking NSAIDs, aniline and salicylic acid and their derivatives, suggesting that depressive symptoms exacerbate pain and its duration (39–41) and may create a vicious cycle of pain and depressive symptoms. Consequently, finding effective therapies for depressed symptoms in patients who also suffer from pain comorbidity is a significant challenge (42). It is uncertain if depression causes pain or whether pain causes depression (43, 44).

However, there are still some limitations of the present study. First, because the data used in this study were primarily conducted among participants of European ancestry, there was a bias toward other ethnic groups with different lifestyles and cultural backgrounds. Second, because only summary-level data were applied in the MR analysis, this limits further stratified analysis of some specific factors, including age, drug use dose and frequency, and other confounding environmental factors. Additionally, although we used large-scale genetic data to obtain instrumental variables for our study, we did not manually check the validity of our instruments through the Phenoscanner. However, we performed sensitivity analyses to assess horizontal pleiotropy and found that our results were robust to potential violations of this assumption. It is possible that our instruments may still be subject to unmeasured confounding or other sources of bias. Finally, one challenge in interpreting our results is the inability to correlate postpartum pain with observed opioid use to postpartum depression. The presence and severity of pain were found to be independently related to the occurrence of depression and anxiety (45), and an increase in the number of pain locations and duration was related to the development of depressed and anxiety symptoms (28). Therefore, the postpartum pain experience and the characteristics of that experience may help us to observe further and study the association between opioids use and the risk of postpartum depression.

## 5. Conclusion

Using MR analysis, our study found that opioids use increases the risk of postpartum depression. We also found that postpartum depression is a potential causal risk factor for increased opioids and three non-opioid analgesics use. Further research is warranted to explore the relationship between postpartum depression and opioids in the development and treatment of postpartum depression.

## Data availability statement

The original contributions presented in the study are included in the article/supplementary material, further inquiries can be directed to the corresponding author.

## Ethics statement

The current Mendelian randomization analysis is based on pooled data and ethical approval were obtained. Secondary analyses of pooled data do not require ethical approval. The patients/participants provided their written informed consent to participate in this study.

## Author contributions

YJ designed the study, collected and analyzed the data, drafted the manuscript, and edited the final manuscript. DW and YX designed the study, collected and analyzed the data, and drafted the manuscript. All authors contributed to the article and approved the submitted version.

## Funding

This study was supported by the National Key Research and Development Program (2018YFC2001905), Guangxi Key Research and Development Program (No. AB20159019), and Guangxi Natural Science Foundation Key Project (2020GXNSFDA238025).

## Conflict of interest

The authors declare that the research was conducted in the absence of any commercial or financial relationships that could be construed as a potential conflict of interest.

## Publisher’s note

All claims expressed in this article are solely those of the authors and do not necessarily represent those of their affiliated organizations, or those of the publisher, the editors and the reviewers. Any product that may be evaluated in this article, or claim that may be made by its manufacturer, is not guaranteed or endorsed by the publisher.
