# Supplementary material for: Causal effects of opioids on postpartum depression: a bidirectional, two-sample Mendelian randomization study
Source: Front Psychiatry. 2023 Apr 20;14:1043854. doi: 10.3389/fpsyt.2023.1043854 (PMC10159056; doi:10.3389/fpsyt.2023.1043854)
Supplement: Supplementary file 1 [file Data_Sheet_1.docx]

Supplementary Material

**Causal Effects of Opioids on Postpartum Depression: A Bidirectional, Two-Sample Mendelian Randomization Study**

Yage Jiang^1,†^, Donglei Wei^1,†^, Yubo Xie^1,2,*^

**^1^ The First Affiliated Hospital of Guangxi Medical University, No. 6 Shuangyong Road, Nanning, Guangxi, China**

**^2^ Department of Anesthesiology, Guangxi Key Laboratory of Enhanced Recovery after Surgery for Gastrointestinal Cancer, Nanning, China.**

**†These authors contributed equally to this work and share first authorship.**

*Correspondence:

Yubo Xie

Email: [xybdoctor@163.com](mailto:xybdoctor@163.com)

**Supplementary tables:**

| **Supplementary Table 1: Genetic Variants (n=25) of Postpartum Depression Used in MR Analyses.** | | | | | | | |
| --- | --- | --- | --- | --- | --- | --- | --- |
| **SNPs** | **Effect allele** | **Other allele** | **Eaf** | **Beta** | **Se** | **pval** | **F** |
| rs10123016 | G | A | 0.3979 | -0.0680 | 0.0145 | 2.97E-06 | 21.84 |
| rs1026186 | A | G | 0.3256 | -0.0701 | 0.0152 | 3.95E-06 | 21.29 |
| rs1249271 | C | G | 0.1924 | -0.0905 | 0.0186 | 1.09E-06 | 23.76 |
| rs12787092 | A | T | 0.1429 | -0.0964 | 0.0209 | 3.87E-06 | 21.33 |
| rs13008867 | C | G | 0.1533 | 0.0971 | 0.0194 | 5.23E-07 | 25.18 |
| rs147968748 | G | A | 0.0110 | 0.3101 | 0.0645 | 1.50E-06 | 23.15 |
| rs186404370 | T | C | 0.0301 | 0.1856 | 0.0407 | 4.98E-06 | 20.85 |
| rs189348675 | C | T | 0.0632 | -0.1417 | 0.0306 | 3.63E-06 | 21.45 |
| rs232475 | A | G | 0.7169 | 0.0772 | 0.0158 | 1.07E-06 | 23.80 |
| rs2347923 | A | C | 0.6771 | -0.0879 | 0.0150 | 4.27E-09 | 34.50 |
| rs3097821 | T | C | 0.4698 | -0.0671 | 0.0143 | 2.54E-06 | 22.14 |
| rs3212737 | C | G | 0.0519 | -0.1685 | 0.0334 | 4.45E-07 | 25.49 |
| rs34595546 | G | A | 0.0585 | -0.1499 | 0.0314 | 1.79E-06 | 22.80 |
| rs35509548 | G | A | 0.3371 | -0.0727 | 0.0151 | 1.49E-06 | 23.16 |
| rs61461427 | A | T | 0.1640 | 0.0866 | 0.0188 | 4.31E-06 | 21.12 |
| rs625274 | T | C | 0.4708 | -0.0666 | 0.0142 | 2.85E-06 | 21.91 |
| rs713763 | A | G | 0.4270 | 0.0667 | 0.0143 | 2.93E-06 | 21.86 |
| rs7157223 | C | T | 0.3259 | 0.0703 | 0.0151 | 3.20E-06 | 21.69 |
| rs7158120 | C | T | 0.6466 | -0.0743 | 0.0147 | 4.62E-07 | 25.42 |
| rs72746111 | C | G | 0.0235 | 0.2303 | 0.0443 | 2.02E-07 | 27.01 |
| rs73371347 | G | A | 0.2653 | -0.0779 | 0.0163 | 1.69E-06 | 22.92 |
| rs75463048 | G | A | 0.0175 | -0.2607 | 0.0566 | 4.08E-06 | 21.23 |
| rs75940076 | A | G | 0.1123 | -0.1056 | 0.0230 | 4.62E-06 | 20.99 |
| rs76209227 | T | A | 0.0239 | 0.2077 | 0.0450 | 3.94E-06 | 21.29 |
| rs77710200 | G | A | 0.0408 | 0.1661 | 0.0347 | 1.65E-06 | 22.96 |
| MR-PRESSO global test pval: 0.1314 | | | | | | | |

| **Supplementary Table 2: Genetic variants (n=31) of opioids used in MR analyses.** | | | | | | | |
| --- | --- | --- | --- | --- | --- | --- | --- |
| **SNPs** | **Effect allele** | **Other allele** | **Eaf** | **Beta** | **Se** | **pval** | **F** |
| rs113544339 | G | T | 0.0232 | -0.1657 | 0.0358 | 3.60E-06 | 21.47 |
| rs11743674 | C | G | 0.2224 | 0.0605 | 0.0129 | 2.60E-06 | 22.07 |
| rs117710046 | T | C | 0.0158 | 0.2097 | 0.0431 | 1.20E-06 | 23.64 |
| rs12238134 | A | G | 0.3051 | -0.0715 | 0.0116 | 8.20E-10 | 37.71 |
| rs12357321 | A | G | 0.3118 | 0.0632 | 0.0117 | 7.10E-08 | 29.05 |
| rs13135092 | G | A | 0.0814 | 0.0987 | 0.0197 | 5.20E-07 | 25.19 |
| rs138200696 | C | A | 0.0162 | 0.1988 | 0.0424 | 2.80E-06 | 21.95 |
| rs138405014 | A | G | 0.0130 | -0.2178 | 0.0475 | 4.60E-06 | 21.01 |
| rs139152977 | A | T | 0.0251 | 0.1638 | 0.0344 | 1.90E-06 | 22.69 |
| rs1397440 | T | C | 0.3251 | -0.0535 | 0.0115 | 3.50E-06 | 21.51 |
| rs140729888 | C | T | 0.0146 | -0.2050 | 0.0446 | 4.30E-06 | 21.11 |
| rs1519369 | T | C | 0.3270 | -0.0533 | 0.0116 | 4.00E-06 | 21.24 |
| rs17487601 | A | G | 0.3565 | -0.0555 | 0.0112 | 7.70E-07 | 24.42 |
| rs2253491 | A | G | 0.2082 | -0.0670 | 0.0132 | 3.60E-07 | 25.88 |
| rs2289739 | T | G | 0.3434 | 0.0543 | 0.0114 | 2.00E-06 | 22.62 |
| rs2297197 | G | C | 0.2808 | 0.0602 | 0.0122 | 7.70E-07 | 24.44 |
| rs4258296 | C | T | 0.3635 | 0.0555 | 0.0112 | 6.50E-07 | 24.76 |
| rs4763505 | T | A | 0.3175 | -0.0531 | 0.0116 | 4.50E-06 | 21.04 |
| rs521956 | T | C | 0.4830 | -0.0538 | 0.0107 | 5.10E-07 | 25.22 |
| rs56226325 | T | C | 0.1547 | -0.0745 | 0.0148 | 4.90E-07 | 25.30 |
| rs56262049 | C | T | 0.1823 | -0.0663 | 0.0141 | 2.50E-06 | 22.20 |
| rs7110825 | C | T | 0.2702 | 0.0570 | 0.0121 | 2.30E-06 | 22.32 |
| rs7154623 | G | A | 0.1932 | 0.0644 | 0.0136 | 2.40E-06 | 22.25 |
| rs7319102 | G | A | 0.2287 | 0.0603 | 0.0128 | 2.40E-06 | 22.22 |
| rs73228014 | A | G | 0.0243 | 0.1610 | 0.0348 | 3.80E-06 | 21.38 |
| rs7428430 | T | C | 0.4826 | -0.0652 | 0.0107 | 1.20E-09 | 37.00 |
| rs76034781 | A | G | 0.0768 | 0.0981 | 0.0202 | 1.20E-06 | 23.52 |
| rs7631941 | T | C | 0.1309 | 0.0730 | 0.0160 | 4.90E-06 | 20.87 |
| rs7860752 | G | A | 0.1927 | 0.0695 | 0.0136 | 3.40E-07 | 26.01 |
| rs945211 | G | C | 0.3834 | -0.0514 | 0.0110 | 3.20E-06 | 21.71 |
| rs975376 | C | G | 0.1643 | 0.0712 | 0.0146 | 1.00E-06 | 23.87 |
| MR-PRESSO global test pval: 0.1360 | | | | | | | |

| **Supplementary Table 3: Genetic variants (n=37) of NSAIDs used in MR analyses.** | | | | | | | |
| --- | --- | --- | --- | --- | --- | --- | --- |
| **SNPs** | **Effect allele** | **Other allele** | **Eaf** | **Beta** | **Se** | **pval** | **F** |
| rs10164103 | G | A | 0.0971 | -0.0576 | 0.0117 | 8.00E-07 | 24.36 |
| rs11097493 | A | T | 0.3606 | 0.0340 | 0.0072 | 2.30E-06 | 22.33 |
| rs11171710 | A | G | 0.4467 | 0.0339 | 0.0070 | 1.40E-06 | 23.26 |
| rs113041162 | A | G | 0.1472 | 0.0499 | 0.0098 | 3.20E-07 | 26.10 |
| rs114212906 | T | C | 0.0469 | 0.0921 | 0.0163 | 1.70E-08 | 31.80 |
| rs11597763 | G | A | 0.2035 | 0.0430 | 0.0086 | 5.30E-07 | 25.14 |
| rs11993835 | A | G | 0.1337 | -0.0489 | 0.0102 | 1.70E-06 | 22.88 |
| rs12285912 | G | A | 0.0263 | 0.1033 | 0.0216 | 1.70E-06 | 22.86 |
| rs12409445 | C | T | 0.1893 | -0.0448 | 0.0090 | 6.80E-07 | 24.66 |
| rs12522598 | G | A | 0.2886 | 0.0452 | 0.0076 | 3.00E-09 | 35.20 |
| rs144713500 | A | G | 0.0130 | 0.1401 | 0.0306 | 4.50E-06 | 21.02 |
| rs1458612 | T | C | 0.3030 | -0.0348 | 0.0075 | 3.70E-06 | 21.41 |
| rs151415 | G | C | 0.2373 | 0.0426 | 0.0082 | 2.10E-07 | 26.98 |
| rs1544861 | T | C | 0.3327 | -0.0366 | 0.0074 | 6.60E-07 | 24.72 |
| rs1589738 | A | G | 0.3677 | 0.0332 | 0.0072 | 3.60E-06 | 21.45 |
| rs1632360 | G | A | 0.0554 | 0.0789 | 0.0152 | 2.00E-07 | 27.01 |
| rs17081175 | A | C | 0.2648 | -0.0381 | 0.0079 | 1.30E-06 | 23.48 |
| rs2237257 | G | T | 0.1229 | -0.0515 | 0.0106 | 1.10E-06 | 23.79 |
| rs2517611 | G | A | 0.2300 | -0.0505 | 0.0082 | 7.10E-10 | 37.99 |
| rs2608029 | G | C | 0.3342 | 0.0362 | 0.0073 | 8.50E-07 | 24.25 |
| rs3001426 | C | T | 0.4528 | -0.0562 | 0.0070 | 1.00E-15 | 64.37 |
| rs34862454 | C | T | 0.3297 | -0.0402 | 0.0073 | 4.40E-08 | 29.97 |
| rs359431 | C | T | 0.4369 | 0.0367 | 0.0070 | 1.30E-07 | 27.84 |
| rs3737240 | T | C | 0.3947 | -0.0368 | 0.0070 | 1.80E-07 | 27.28 |
| rs3821269 | A | G | 0.4984 | 0.0375 | 0.0069 | 5.90E-08 | 29.39 |
| rs4110942 | G | A | 0.0877 | -0.0564 | 0.0122 | 3.90E-06 | 21.31 |
| rs533650768 | G | A | 0.0179 | 0.1263 | 0.0262 | 1.50E-06 | 23.17 |
| rs55842862 | C | G | 0.0749 | 0.0629 | 0.0131 | 1.60E-06 | 23.00 |
| rs56044130 | G | A | 0.0946 | 0.0580 | 0.0118 | 9.40E-07 | 24.05 |
| rs56166763 | C | G | 0.3686 | -0.0409 | 0.0072 | 1.20E-08 | 32.53 |
| rs56236914 | T | C | 0.1844 | 0.0486 | 0.0089 | 5.50E-08 | 29.53 |
| rs6891880 | G | A | 0.4272 | -0.0385 | 0.0071 | 4.90E-08 | 29.74 |
| rs71510292 | C | T | 0.0480 | 0.0817 | 0.0163 | 5.40E-07 | 25.13 |
| rs7532754 | T | C | 0.3598 | -0.0343 | 0.0072 | 1.70E-06 | 22.85 |
| rs7687973 | C | T | 0.2245 | -0.0443 | 0.0083 | 1.00E-07 | 28.38 |
| rs7856204 | C | G | 0.3637 | 0.0344 | 0.0072 | 1.80E-06 | 22.84 |
| rs8013531 | T | C | 0.4055 | 0.0340 | 0.0071 | 1.40E-06 | 23.25 |
| MR-PRESSO global test pval: 0.2254 | | | | | | | |

| **Supplementary Table 4: Genetic variants (n=32) of Salicylic Acids Used in MR Analyses.** | | | | | | | | |
| --- | --- | --- | --- | --- | --- | --- | --- | --- |
| **SNPs** | **Effect allele** | **Other allele** | **Eaf** | **Beta** | **Se** | **pval** | **F** |  |
| rs10218528 | A | T | 0.3931 | -0.0411 | 0.0082 | 4.50E-07 | 25.45 |  |
| rs10508267 | C | G | 0.3595 | -0.0393 | 0.0084 | 2.70E-06 | 22.01 |  |
| rs10887793 | T | G | 0.4482 | 0.0427 | 0.0080 | 1.00E-07 | 28.30 |  |
| rs11206268 | G | A | 0.0658 | -0.0754 | 0.0159 | 2.20E-06 | 22.38 |  |
| rs113242618 | A | G | 0.0144 | -0.1542 | 0.0332 | 3.40E-06 | 21.59 |  |
| rs113551213 | A | G | 0.0495 | 0.0851 | 0.0185 | 4.10E-06 | 21.21 |  |
| rs117571676 | A | T | 0.0198 | -0.1332 | 0.0285 | 3.10E-06 | 21.78 |  |
| rs117733303 | G | A | 0.0197 | 0.1846 | 0.0285 | 8.90E-11 | 42.05 |  |
| rs11874619 | C | G | 0.3876 | -0.0386 | 0.0083 | 3.30E-06 | 21.61 |  |
| rs12188010 | A | T | 0.3756 | 0.0398 | 0.0082 | 1.20E-06 | 23.60 |  |
| rs12580718 | A | G | 0.3668 | -0.0391 | 0.0083 | 2.20E-06 | 22.38 |  |
| rs139696858 | A | G | 0.0136 | -0.1642 | 0.0342 | 1.60E-06 | 23.05 |  |
| rs1960743 | A | G | 0.1093 | 0.0586 | 0.0127 | 4.00E-06 | 21.26 |  |
| rs2523589 | T | G | 0.4952 | 0.0444 | 0.0079 | 2.10E-08 | 31.39 |  |
| rs28601761 | G | C | 0.4151 | -0.0607 | 0.0082 | 1.30E-13 | 54.84 |  |
| rs4299376 | G | T | 0.3244 | 0.0453 | 0.0085 | 8.60E-08 | 28.66 |  |
| rs4995008 | A | G | 0.1309 | 0.0541 | 0.0118 | 4.30E-06 | 21.13 |  |
| rs56226325 | T | C | 0.1548 | -0.0567 | 0.0109 | 2.20E-07 | 26.86 |  |
| rs583104 | G | T | 0.2247 | -0.0780 | 0.0095 | 1.70E-16 | 67.95 |  |
| rs59681006 | C | T | 0.1299 | -0.0566 | 0.0118 | 1.70E-06 | 22.87 |  |
| rs635634 | T | C | 0.1845 | 0.0666 | 0.0102 | 7.40E-11 | 42.40 |  |
| rs7244655 | A | G | 0.2483 | -0.0433 | 0.0092 | 2.20E-06 | 22.38 |  |
| rs73015016 | A | G | 0.1177 | -0.0957 | 0.0123 | 6.40E-15 | 60.77 |  |
| rs7315004 | A | T | 0.4187 | -0.0408 | 0.0081 | 4.60E-07 | 25.42 |  |
| rs7412 | T | C | 0.0784 | -0.1108 | 0.0147 | 4.90E-14 | 56.77 |  |
| rs74617384 | T | A | 0.0827 | 0.1050 | 0.0144 | 2.70E-13 | 53.41 |  |
| rs75278536 | G | T | 0.1064 | -0.0673 | 0.0128 | 1.60E-07 | 27.45 |  |
| rs77507211 | T | G | 0.0179 | 0.1365 | 0.0299 | 4.80E-06 | 20.91 |  |
| rs896232 | T | C | 0.3613 | -0.0395 | 0.0082 | 1.50E-06 | 23.18 |  |
| rs950100 | A | G | 0.0691 | -0.0715 | 0.0156 | 4.50E-06 | 21.06 |  |
| rs964184 | G | C | 0.1339 | 0.0633 | 0.0116 | 4.90E-08 | 29.77 |  |
| rs9982261 | A | G | 0.0113 | -0.1749 | 0.0376 | 3.30E-06 | 21.62 |  |
| MR-PRESSO global test pval: 0.5034 | | | | | | | | |

| **Supplementary Table 5: Genetic variants (n=37) of anilides used in MR analyses.** | | | | | | | |
| --- | --- | --- | --- | --- | --- | --- | --- |
| **SNPs** | **Effect allele** | **Other allele** | **Eaf** | **Beta** | **Se** | **pval** | **F** |
| rs10033431 | C | T | 0.2669 | 0.0376 | 0.0074 | 4.10E-07 | 25.66 |
| rs11172113 | C | T | 0.4097 | -0.0534 | 0.0066 | 8.90E-16 | 64.66 |
| rs11242905 | C | T | 0.1270 | 0.0452 | 0.0098 | 4.30E-06 | 21.14 |
| rs12029331 | T | C | 0.4152 | 0.0322 | 0.0066 | 1.30E-06 | 23.48 |
| rs12438004 | C | A | 0.4189 | 0.0352 | 0.0067 | 1.30E-07 | 27.84 |
| rs12568655 | A | G | 0.3883 | -0.0407 | 0.0067 | 1.20E-09 | 37.03 |
| rs1544115 | T | C | 0.2469 | 0.0350 | 0.0076 | 4.30E-06 | 21.12 |
| rs1648368 | T | C | 0.1266 | -0.0467 | 0.0098 | 2.00E-06 | 22.61 |
| rs17191307 | G | T | 0.2538 | 0.0355 | 0.0075 | 2.20E-06 | 22.42 |
| rs17449582 | T | C | 0.3642 | 0.0342 | 0.0068 | 4.20E-07 | 25.60 |
| rs17652520 | A | G | 0.2314 | 0.0557 | 0.0078 | 8.10E-13 | 51.26 |
| rs1942262 | A | G | 0.2927 | 0.0369 | 0.0072 | 3.00E-07 | 26.25 |
| rs2274319 | T | C | 0.3482 | 0.0360 | 0.0069 | 1.50E-07 | 27.62 |
| rs2430157 | T | C | 0.4729 | -0.0308 | 0.0065 | 2.60E-06 | 22.10 |
| rs2526922 | A | G | 0.4716 | -0.0304 | 0.0065 | 3.50E-06 | 21.53 |
| rs2825128 | C | A | 0.4463 | 0.0314 | 0.0066 | 1.90E-06 | 22.69 |
| rs2930291 | A | G | 0.3626 | -0.0320 | 0.0068 | 2.60E-06 | 22.11 |
| rs293566 | C | T | 0.3327 | 0.0347 | 0.0069 | 5.70E-07 | 25.01 |
| rs3130486 | T | C | 0.2652 | -0.0549 | 0.0074 | 1.00E-13 | 55.30 |
| rs41272663 | A | C | 0.2624 | -0.0350 | 0.0074 | 2.40E-06 | 22.21 |
| rs4356969 | C | G | 0.2640 | -0.0363 | 0.0075 | 1.40E-06 | 23.32 |
| rs4663983 | G | A | 0.1920 | -0.0558 | 0.0083 | 1.70E-11 | 45.31 |
| rs4718618 | A | G | 0.1851 | 0.0395 | 0.0084 | 2.70E-06 | 22.00 |
| rs4741328 | C | T | 0.3681 | -0.0330 | 0.0068 | 1.10E-06 | 23.74 |
| rs59357103 | A | G | 0.1663 | 0.0410 | 0.0088 | 3.30E-06 | 21.65 |
| rs62180637 | C | T | 0.4662 | -0.0313 | 0.0066 | 1.80E-06 | 22.76 |
| rs62378567 | G | A | 0.0194 | -0.1156 | 0.0237 | 1.00E-06 | 23.87 |
| rs6568392 | G | T | 0.2190 | 0.0402 | 0.0079 | 3.60E-07 | 25.92 |
| rs73168813 | C | G | 0.0217 | 0.1063 | 0.0224 | 2.10E-06 | 22.49 |
| rs73531210 | T | C | 0.0507 | -0.0699 | 0.0149 | 2.50E-06 | 22.14 |
| rs73998427 | T | C | 0.0315 | 0.0875 | 0.0187 | 2.90E-06 | 21.90 |
| rs74618578 | G | A | 0.0260 | -0.1037 | 0.0206 | 4.60E-07 | 25.42 |
| rs75036981 | A | G | 0.0613 | 0.0659 | 0.0137 | 1.50E-06 | 23.18 |
| rs7567892 | T | C | 0.0988 | 0.0614 | 0.0110 | 2.00E-08 | 31.46 |
| rs9571576 | C | T | 0.4942 | 0.0318 | 0.0066 | 1.20E-06 | 23.60 |
| rs9915717 | A | G | 0.2031 | 0.0417 | 0.0082 | 3.10E-07 | 26.17 |
| rs9925184 | C | T | 0.4358 | 0.0328 | 0.0067 | 8.10E-07 | 24.32 |
| MR-PRESSO global test pval: 0.2812 | | | | | | | |
